# Cytokines Alter IgA1 *O*-Glycosylation by Dysregulating C1GalT1 and ST6GalNAc-II Enzymes*

**DOI:** 10.1074/jbc.M113.512277

**Published:** 2013-01-07

**Authors:** Hitoshi Suzuki, Milan Raska, Koshi Yamada, Zina Moldoveanu, Bruce A. Julian, Robert J. Wyatt, Yasuhiko Tomino, Ali G. Gharavi, Jan Novak

**Affiliations:** From the ‡ University of Alabama at Birmingham, Birmingham, Alabama 35294,; § Juntendo University Faculty of Medicine, Tokyo 113-8421, Japan,; ¶ Palacky University Olomouc, Olomouc 779 00, Czech Republic,; ‖ University of Tennessee Health Science Center, Memphis, Tennessee 38103, and; ** Columbia University, New York, New York 10032

**Keywords:** Glycosylation, Immunology, Kidney, Mucosal Immunology, Nephrology, IgA Nephropathy, IgA1, O-Glycans

## Abstract

IgA nephropathy (IgAN), the most common primary glomerulonephritis, is characterized by renal immunodeposits containing IgA1 with galactose-deficient *O*-glycans (Gd-IgA1). These immunodeposits originate from circulating immune complexes consisting of anti-glycan antibodies bound to Gd-IgA1. As clinical disease onset and activity of IgAN often coincide with mucosal infections and dysregulation of cytokines, we hypothesized that cytokines may affect IgA1 *O*-glycosylation. We used IgA1-secreting cells derived from the circulation of IgAN patients and healthy controls and assessed whether IgA1 *O*-glycosylation is altered by cytokines. Of the eight cytokines tested, only IL-6 and, to a lesser degree, IL-4 significantly increased galactose deficiency of IgA1; changes in IgA1 *O*-glycosylation were robust for the cells from IgAN patients. These cytokines reduced galactosylation of the *O*-glycan substrate directly via decreased expression of the galactosyltransferase C1GalT1 and, indirectly, via increased expression of the sialyltransferase ST6GalNAc-II, which prevents galactosylation by C1GalT1. These findings were confirmed by siRNA knockdown of the corresponding genes and by *in vitro* enzyme reactions. In summary, IL-6 and IL-4 accentuated galactose deficiency of IgA1 via coordinated modulation of key glycosyltransferases. These data provide a mechanism explaining increased immune-complex formation and disease exacerbation during mucosal infections in IgAN patients.

## Teaser

**Background:** IgA1-producing cells of IgA nephropathy patients secrete IgA1 with galactose-deficient *O*-glycans; these IgA1 molecules form nephritogenic immune complexes.

**Results:** Cytokines IL-6 and IL-4 accentuate IgA1 galactose deficiency via modulation of key glycosyltransferases.

**Conclusion:** Some cytokines alter IgA1 *O*-glycosylation and increase production of nephritogenic IgA1 glycoforms.

**Significance:** These data provide a mechanism explaining increased immune-complex formation in IgA nephropathy patients.

## Introduction

IgA nephropathy (IgAN)^3^ is the most common primary glomerulonephritis world-wide and an important cause of renal failure (1, 2). IgAN is characterized by mesangial IgA-containing immune deposits, often with IgG and/or IgM codeposits (3–6). IgAN manifests most frequently in adolescents and young adults, and asymptomatic proteinuria and hematuria are characteristic clinical presentations (2, 7, 8). Macroscopic hematuria in IgAN patients often coincides with mucosal infections, including infections of the upper respiratory tract and/or digestive system, suggesting an environmental contributing factor(s).

The IgA in the glomerular deposits is exclusively of the IgA1 subclass (9). IgA1 contains a hinge region in its heavy chain that is the site of attachment of 3–6 *O*-glycans (10–17). Normal human IgA1 in the circulation has core 1 *O*-glycans consisting of *N*-acetylgalactosamine (GalNAc) with a β1,3-linked galactose; each saccharide may be sialylated (12, 18–22). Normal serum IgA1 is thought to contain little or no galactose-deficient *O*-glycans (12). In contrast, patients with IgAN have elevated levels of circulatory IgA1 with some galactose-deficient *O*-glycans, *i.e.* consisting of terminal GalNAc or sialylated GalNAc (23–28). Although the glycosylation of IgA1 is altered, that of other glycoproteins with *O*-glycans is not (24, 29). Moreover, the increased serum level of galactose-deficient IgA1 (Gd-IgA1) is a hereditable trait, suggesting a genetic co-determination factor(s) in the pathogenesis of IgAN (30).

IgA1 with galactose-deficient *O*-glycans is a pivotal feature of IgAN (31–33). Multiple observations support this conclusion, including the fact that IgA1 in the glomerular immunodeposits is enriched for galactose-deficient glycoforms (34, 35). This glomerular IgA1 arises from deposition of immune complexes from the circulation and/or *in situ* binding of anti-glycan antibodies to deposited Gd-IgA1 (26, 32, 33, 36–38). In the mesangium, these IgA1-containing immune complexes activate resident mesangial cells and thereby stimulate their proliferation and overproduction of extracellular matrix (39–44), leading to glomerular injury (45, 46).

Generation of immortalized IgA1-secreting cells derived from the circulation of patients with IgAN and healthy and disease controls and analyses of the secreted IgA1 have provided insight as to the origin of Gd-IgA1. Cells from IgAN patients secrete IgA1 for which the degree of galactose deficiency mimics that of circulatory IgA1 from the corresponding donors (47). The IgA1 galactose deficiency is related to decreased expression and activity of core 1 β1,3-galactosyltransferase (C1GalT1; encoded by *C1GALT1* gene) (48) that adds galactose to GalNAc and elevated expression and activity of α-*N*-acetylgalactosaminide α-2,6-sialyltransferase 2 (ST6GalNAc-II; encoded by *ST6GALNAC2* gene) (49) that adds sialic acid to GalNAc (47). Moreover, the expression of C1GalT1-specific chaperone (encoded by *COSMC* gene) (50, 51) necessary for stability of the nascent C1GalT1 protein is decreased (47).

Infections, such as those associated with synpharyngitic visible hematuria in IgAN patients, may alter production of multiple cytokines. For example, serum levels of TNF and IL-6 are elevated in IgAN patients (52). Moreover, some cytokines are known to affect immunoglobulin glycosylation. In mice, Th2 cytokines IL-4 and IL-5 alter *N*-glycosylation of murine IgA (53). As mice have only one subclass of IgA that generally does not have *O*-glycans, another group tested the effect of Th2 cytokines using human IgA1 cell line Dakiki and found that IL-4 increases galactose deficiency of the secreted IgA1 (54). However, none of these studies used IgA1-producing cells from IgAN patients.

To assess possible relevance to IgAN, we used our panel of immortalized IgA1-producing cells from IgAN patients and controls and tested the effect of selected cytokines on IgA1 glycosylation. These experiments revealed that IL-6 and, to a lesser extent, IL-4 increased galactose deficiency of IgA1 secreted by the cells from IgAN patients. We showed that the mechanisms of the IL-6-enhanced aberrant glycosylation of IgA1 involved dysregulated expression and activity of two key glycosyltransferases, elevated for ST6GalNAc-II and decreased for C1GalT1. Moreover, premature sialylation of Gd-IgA1 *O*-glycans prevented effective galactosylation of GalNAc by C1GalT1. These conclusions were confirmed by siRNA knockdown of the corresponding genes and by *in vitro* enzyme reactions. Together, these results reveal the role of abnormal cytokine responses that enhance synthesis of Gd-IgA1 in IgA1-producing cells from patients with IgAN and identify potential targets for disease-specific intervention of this chronic disease.

## MATERIALS AND METHODS

### 

#### 

##### Patients and Controls

This study was approved by University of Alabama at Birmingham Institutional Review Board and the Ethical Review Board of Juntendo University Hospital. IgA1-secreting cells and the donors have been described previously (47). Patients with IgAN (*n* = 23) were recruited at Juntendo University Hospital. Serum samples were collected at the time of renal biopsy. Serum samples were also collected from 11 healthy volunteers. Characteristics of these subjects are described in Table 1.

##### Cell Cultures and Cytokine Treatment

B cells from the blood of patients with IgAN and healthy controls had been immortalized with Epstein-Barr virus, subcloned to obtain clones of IgA1-producing cell lines, and cultured as described (47). Cells were maintained in culture for 7 days. Cytokines were purchased from R&D Systems (Minneapolis, MN) and used at concentrations established in previous studies: IL-1 (10 pg/ml), IL-4 (2 ng/ml), IL-5 (2 ng/ml), IL-6 (8 ng/ml), IL-10 (7.5 ng/ml), IFNγ (7.5 ng/ml), TGFβ (2 ng/ml), and TNFα (1 ng/ml) (53–64). The control cultures did not have any cytokine added.

##### Measurement of Galactose-deficient IgA1

We used a protocol described previously in detail (15, 47). Briefly, F(ab′)_2_ fragment of goat anti-human IgA (Jackson ImmunoResearch Laboratories, West Grove, PA) was coated onto ELISA plates. Samples in serial dilutions were applied, and the captured IgA was treated with neuraminidase to remove sialic acid residues. After washing, the samples were reacted with biotin-labeled GalNAc-specific lectin isolated from *Helix aspersa* (HAA; Sigma) followed by HRP-avidin and peroxidase substrate. The absorbance was measured at 490 nm and expressed in units; 100 units was defined as HAA binding to 50 ng of standard Gd-IgA1 (Ale) myeloma protein.

##### Enzyme Activities of C1GalT1 and ST6GalNAc-II

Analysis of C1GalT1 and ST6GalNAc-II activities in cell lines stimulated with IL-4 or by IL-6 was performed using Golgi-enriched enzyme preparations isolated as described (47, 49). Briefly, the sialyltransferase assay (ST6GalNAc-II) was performed using CMP-*N*-acetylneuraminic acid as the sialic-acid donor and enzymatically desialylated IgA1 (Mce) myeloma protein as the acceptor (47, 49). The galactosyltransferase assay (C1GalT1) was performed with UDP-galactose as the galactose donor and enzymatically desialylated IgA1 (Mce) myeloma protein as the acceptor (47). The reactions were performed for 6 h at 37 °C with gentle shaking.

To test whether prior sialylation inhibits subsequent galactosylation, GalNAc on Gd-IgA1 was sialylated by sialyltransferase in the Golgi-enriched preparations from IgA1-secreting cells from IgAN patients and was then subjected to a galactosylation reaction with the enzyme in the Golgi-enriched preparations from IgA1-secreting cells from IgAN patients or from healthy controls. Briefly, 2 μg of desialylated Gd-IgA1 (Ale) myeloma protein served as acceptor and 100 mm CMP-*N*-acetylneuraminic acid served as the sialic-acid donor with 10 μl of Golgi-enriched fractions from 10 × 10^6^ IgA1-producing cells from IgAN patients as the source of ST6GalNAc-II (47). The reaction was performed for 4 h at 37 °C with gentle shaking. Then, buffer was changed to PBS using ultrafiltration (30-kDa cut-off ultrafilters, Millipore, Billerica, MA). Subsequently, C1GalT1 reaction was performed with 10 μl of Golgi-enriched fraction from 10 × 10^6^ IgA1-producing cells from IgAN patients or from healthy controls as the enzyme source and 0.4 mm UDP-galactose as the galactose donor (47). The reaction was performed for 4 h at 37 °C with gentle shaking. After the reactions, HAA binding to IgA1 proteins was measured using normalized IgA1 amounts in lectin ELISA with and without neuraminidase treatment (47).

##### Real Time RT-PCR Analysis

RNA was isolated using RNAStat60 or RNeasy 96 Mini kit (Qiagen, Valencia, CA) converted to cDNA by the use of SuperScript III First-Strand Synthesis SuperMix kit (Invitrogen) according to the manufacturer's instructions. Levels of *C1GALT1*, *COSMC*, and *ST6GALNAC2* transcripts were determined by real time RT-PCR using LightCycler 480 DNA SYBR Green I Master on LightCycler 480 instrument (Roche Applied Science) and expressed relative to the expression of β-actin or the *GAPDH* housekeeping gene (47).

##### C1GALT1 and ST6GALNAC2 siRNA Treatment

EBV-immortalized cell lines from three patients with IgAN and three healthy individuals were transfected using ON-TARGETplus SMARTpool siRNAs (Thermo Fisher Scientific, Lafayette, CO) specific for human *C1GALT1* or *ST6GALNAC2*. The ON-TARGETplus Non-targeting Pool siRNAs was used as a control. In brief, cells were cultured for 24 h in RPMI 1640 (Invitrogen), 20% FBS (Invitrogen), and 1× Pen-Strep (Atlanta Biologicals, Lawrenceville, GA) at a density 5 × 10^5^/ml. Before transfection, the cells were harvested by centrifugation at 300 × *g* for 10 min and resuspended at room temperature in Nucleofector Solution C (Lonza, Cologne, Germany) at a density of 2.5 × 10^6^/100 μl for each transfection. After the addition of 1.4 μg of individual siRNA, cells were pulsed in Amaxa nucleofector II (Lonza, Allendale, NJ) using program X-001, immediately transferred to a 24-well panel containing 1.4 ml of RPMI 1640 medium with 20% FBS and antibiotics, and incubated in humidified CO_2_ incubator at 37 °C. Twenty-four hours after transfection, the knockdown efficiency was determined by real time RT-PCR as described above. The knockdown was expressed as the cDNA level of the individual gene normalized to GAPDH after respective siRNA treatment, divided by respective value obtained after treatment by non-targeting siRNA.

##### SDS-PAGE/Western Blot Analyses

Molecular forms of IgA1 secreted in the culture media by IgA1-producing cells were assessed by immunoblotting after SDS-PAGE under non-reducing conditions. Blots were developed with polyclonal anti-IgA1 biotinylated antibody and detected with HRP-neutravidin using SuperSignal West Pico reagents (Pierce) (47).

##### Statistics

Correlations between different parameters were analyzed by Student's *t* test, two-tailed, or by the Mann-Whitney test. Analysis of variance was used to determine differences in the characteristics among multiple groups. Data were expressed as the mean ± S.D. or median values. *p* values less than 0.05 were considered significant. All statistical analyses were performed with StatView 5.0 software (Abacus Concept Inc., Berkeley, CA).

## RESULTS

### 

#### 

##### IL-6 or IL-4 Stimulation of IgA1-producing Cells from IgAN Patients Accentuates Galactose Deficiency of IgA1 O-Glycans

**FIGURE 1.**
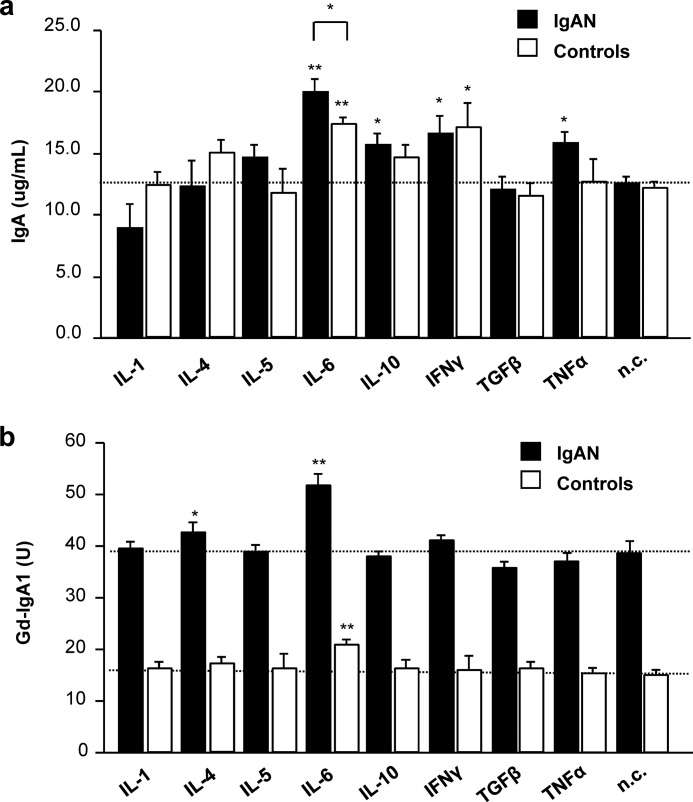
**Influence of cytokines on IgA1 production and *O*-glycosylation by IgA1-secreting cells.** Cell lines from three IgAN patients (IgAN; *black columns*) and three healthy controls (*white columns*, controls) were used. *a*, IgA production. The *horizontal dashed line* marks the mean IgA concentration for non-stimulated cells from IgAN patients (*n.c.*). *b*, production of galactose-deficient IgA1 (Gd-IgA1). *Horizontal dashed lines* mark the mean Gd-IgA1 concentrations from non-stimulated cells (*n.c.*) from IgAN patients (*upper line*) and healthy controls (*lower line*). *U*, units. 100 units was defined as HAA binding to 50 ng of standard Gd-IgA1 (Ale) myeloma protein. Mean values ± S.D. from two independent experiments are shown. *n.c.*, negative control (*i.e.* not stimulated with any cytokine); *, *p* < 0.05; **, *p* < 0.01 for comparisons with the negative control.

We tested the effects of cytokine supplementation on IgA1 production and *O*-glycosylation using cultured immortalized IgA1-secreting cells from patients with IgAN and healthy controls. We used IL-1, IL-4, IL-5, IL-6, IL-10, IFNγ, TGFβ, and TNFα. Several cytokines, IL-6, IL-10, IFNγ, and TNFα, increased production of IgA1 in one or both types of cells (*p* < 0.05), but IL-6 showed the greatest effect (*p* < 0.01; Fig. 1*a*). The response to IL-6 was more pronounced for the cells from IgAN patients than that for the cells from the healthy controls (*p* < 0.05). Notably, IL-6 and, to a lesser degree, IL-4 increased galactose deficiency of secreted IgA1 (Fig. 1*b*). The increase in galactose deficiency induced by IL-6 was robust for IgA1 secreted by cells from IgAN patients (increase from 38 to 52 units; *p* < 0.01). The effect of IL-6 on IgA1 secreted by the control cells was also significant (increase from 15 to 21 units; *p* < 0.01), although the degree of galactose deficiency did not reach that of IgA1 produced by cells from IgAN patients. There was no additive effect of IL-6 and IL-4 observed on Gd-IgA1 production by the cells from IgAN patients or controls (data not shown).

Our previous studies revealed that although our panel of IgA1-producing cell lines from IgAN patients secreted mixtures of three molecular forms of IgA1 (monomeric, dimeric, and trimeric), only the polymeric (dimeric and trimeric) forms were galactose-deficient (38). Therefore, we tested whether any of the cytokines may have altered the ratio of the molecular forms of the secreted IgA1. SDS-PAGE under non-reducing conditions followed by Western blot analysis revealed that none of the cytokines altered the ratio of the three molecular forms, indicating that IL6-mediated galactose deficiency was not due to increased production of polymeric IgA1 (data not shown).

##### Cytokines That Increase Galactose Deficiency of IgA1 O-Glycans in Cells from IgAN Patients Alter Expression and Activity of Specific Glycosyltransferases

**FIGURE 2.**
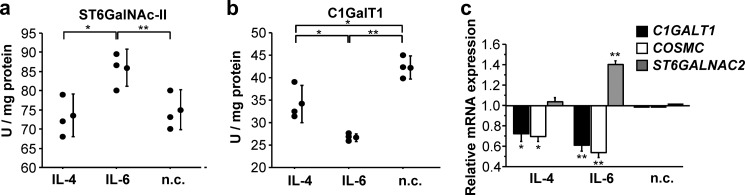
**Changes in enzymatic activity (*a* and *b*) of C1GalT1 and α2,6-GalNAc-sialyltransferase and transcription (*c*) of *C1GALT1, COSMC,* and *ST6GALNAC2* genes in IgA1-secreting cells from three IgAN patients after cytokine stimulation.** Mean values ± S.D. from two experiments are shown. IL-6 increased ST6GalNAc-II activity (*p* < 0.05; *a*) and decreased C1GalT1 activity (*p* < 0.01; *b*). IL-4 did not change ST6GalNAc-II activity (*a*) but reduced C1GalT1 activity (*p* < 0.05; *b*). In *c*), expression of each gene (*C1GALT1, COSMC,* and *ST6GALNAC2*) without cytokine treatment (*n.c.*) is set to 1. **, *p* < 0.01; *, *p* < 0.05.

We measured the activities of key glycosyltransferases, C1GalT1 (encoded by *C1GALT1* gene) and ST6GalNAc-II (encoded by *ST6GALNAC2* gene) (47, 49), using *in vitro* enzyme assays with Golgi-enriched preparations from different cell lines as the source of enzymes. IL-6 treatment increased ST6GalNAc-II activity (*p* < 0.05; Fig. 2*a*) and decreased C1GalT1 activity (*p* < 0.01; Fig. 2*b*). For comparison, we also assessed the effect of IL-4 on activities of both enzymes and found no change in the ST6GalNAc-II activity (Fig. 2*a*) but reduced C1GalT1 activity (*p* < 0.05; Fig. 2*b*). Using quantitative real time RT-PCR, we found that IL-6 increased the expression of *ST6GALNAC2* gene and reduced the expression of *C1GALT1* and *COSMC* genes (*p* < 0.01), in accordance with the measured changes in enzymatic activities. In contrast, IL-4 reduced the expression of only *C1GALT1* and *COSMC* genes, whereas the expression of *ST6GALNAC2* remained unaffected, again in agreement with measured changes in enzymatic activities (*p* < 0.05; Fig. 2*c*).

Next, we analyzed the degree of GalNAc sialylation of *O*-glycans on IgA1 secreted from IL-6- or IL-4-treated cells by the reactivity with HAA (28, 47, 65, 66); HAA lectin reacts with only terminal, not sialylated, GalNAc (47). Therefore, differences in reactivities of HAA with IgA1 treated with neuraminidase, to remove sialic acid, *versus* not treated provides information about relative GalNAc sialylation. HAA reactivity of IgA1 secreted by the cells from IgAN patients increased after neuraminidase treatment (from 21 to 38 units; *p* < 0.05). HAA reactivity of IgA1 secreted by the cells from the controls also increased (from 12 to 16 units; *p* < 0.05) but did not reach the baseline HAA reactivity with IgA1 from cells of IgAN patients (data not shown). These results suggested an extensive sialylation of terminal GalNAc on Gd-IgA1 secreted by cells from IgAN patients. Moreover, IL-6 further increased sialylation of terminal GalNAc on Gd-IgA1 (*p* < 0.01). These findings fit well with the elevated activity of ST6GalNAc-II in cells of IgAN patients that is further augmented by IL-6 stimulation.

##### siRNA Knockdown Experiments Confirm Involvement of C1GalT1 and ST6GalNAc-II in Aberrant O-Glycosylation of IgA1

**FIGURE 3.**
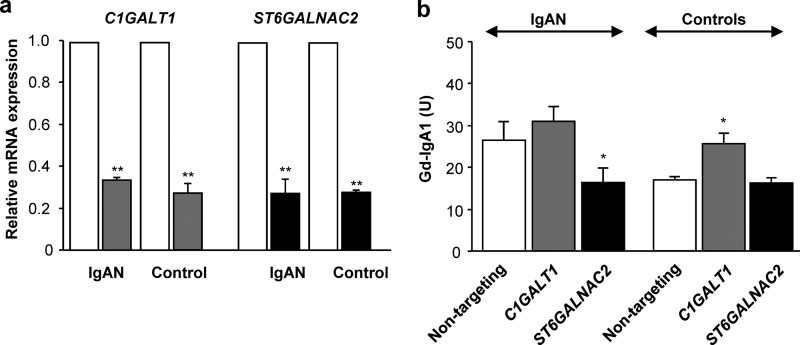
**Knockdown of gene expression of *C1GALT1* or *ST6GALNAC2* by siRNA treatment (*a*) and production of Gd-IgA1 (*b*) by cell lines from patients with IgAN and healthy controls.** *a*, real time RT-PCR analysis of *C1GALT1* or *ST6GALNAC2* confirmed robust knockdown, 65–75% (*i.e.* about two PCR-cycle differences between non-targeting and targeting siRNA). Expression of each gene is shown for mock-control samples (transfection with non-targeting siRNA; *white columns*), *C1GALT1* siRNA treatment (*gray columns*), and *ST6GALNAC2* siRNA treatment (*black columns*). Expression of each gene in mock-control samples was set to 1 for each cell type. Cell lines from three IgAN patients (*IgAN*) and three healthy controls (*Control*) were used. Mean values ± S.D. are shown. **, *p* < 0.01 for comparisons with non-targeting siRNA. *b*, production of Gd-IgA1 secreted by IgA1-producing cells is shown in units as mean values ± S.D. from two independent experiments. *, *p* < 0.05 for comparisons with non-targeting siRNA.

The previous experiments indicated a role for *C1GALT1* and/or *ST6GALNAC2* gene products in the synthesis of Gd-IgA1 in the cells of IgAN patients. To confirm this conclusion, we used siRNA knockdown in IgA1-secreting cells from IgAN patients and healthy controls. Real time RT-PCR analysis revealed that nucleofection-driven transfection with gene-specific siRNA reduced the mRNA level of each gene in all cell types by 65–75% (Fig. 3*a*). *C1GALT1* knockdown increased galactose deficiency in IgA1 secreted by cells from IgAN patients as well as by cells from healthy controls (Fig. 3*b*, *p* < 0.05). In contrast, *ST6GALNAC2* knockdown decreased galactose deficiency in IgA1 secreted by cells from only the IgAN patients (Fig. 3*b*, *p* < 0.05). After the *ST6GALNAC2* knockdown, galactose content on IgA1 secreted by the cells from IgAN patients was comparable with that on IgA1 secreted by the cells from healthy controls. These experiments together directly confirmed the roles of both enzymes, C1GalT1 and ST6GalNAc-II, in the production of Gd-IgA1.

##### Premature Sialylation Blocks Subsequent Galactosylation of Galactose-deficient IgA1 O-Glycans

**FIGURE 4.**
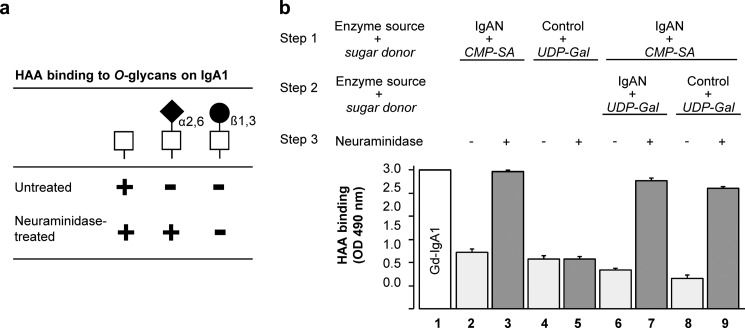
**Premature sialylation blocks enzymatic addition of galactose to IgA1 *O*-glycans.** *a*, GalNAc-specific lectin from HAA binds terminal GalNAc on Gd-IgA1 but not sialylated or galactosylated GalNAc. However, neuraminidase removes sialic acid from sialylated GalNAc that then becomes available for HAA binding. Galactose on GalNAc is not affected by neuraminidase. *b*, Gd-IgA1 myeloma protein binds HAA (*Gd-IgA1*; *column 1*). GalNAc residues on this IgA1 protein were enzymatically sialylated using CMP-*N*-acetylneuraminic acid as the donor (*CMP-SA*; *columns 2* and *3*) or galactosylated using UDP-galactose as the donor (*UDP-Gal*; *columns 4* and *5*) by the corresponding enzymes in the Golgi-enriched preparations from IgA1-secreting cells from IgAN patients (*IgAN*) or from healthy controls (*Control*). HAA binding to these preparations was measured before (−) or after (+) neuraminidase treatment (*Neuraminidase*). These control experiments confirmed that both enzymatic reactions reduced HAA binding (*columns 2* and *4*) and that neuraminidase treatment recovered HAA binding to the enzymatically sialylated (*column 3*) but not to the galactosylated (*column 5*) IgA1 protein. Next, we tested whether prior sialylation prevents subsequent galactosylation. GalNAc on IgA1 (Ale) myeloma protein was sialylated by ST6GalNAc-II in the Golgi-enriched preparations from IgA1-secreting cells from IgAN patients (*Step 1*) and then subjected to a galactosylation reaction (*Step 2*) with C1GalT1 in the Golgi-enriched preparations from IgA1-secreting cells from IgAN patients (IgAN; *columns 6* and *7*) or from healthy controls (*Control*; *columns 8* and *9*). HAA binding to these preparations was measured before (−) or after (+) neuraminidase treatment (*columns 6–9*). These experiments revealed that pre-sialylation of GalNAc significantly reduced subsequent galactosylation even in the presence of normal levels of C1GalT1 in the cells from healthy controls (*columns 8* and *9*). Mean values ± S.D. from two experiments with Golgi-enriched preparations from four different cell lines are shown. *p* < 0.001 for *columns 2 versus 3*, *6 versus 7*, and *8 versus 9*; *p* < 0.01 for *columns 3 versus 7* and *3 versus 9*.

The results of knockdown of *ST6GALNAC2* in the cells from IgAN patients pointed to a specific role of the encoded sialyltransferase, as the knockdown increased the content of galactose on IgA1 secreted by cells from IgAN patients to that on IgA1 from cells of healthy controls (Fig. 3*b*). To explain the observation, we hypothesized that premature sialylation of GalNAc on IgA1 may inhibit subsequent galactosylation. To experimentally test this hypothesis, we developed an *in vitro* assay based on the assumptions that (i) the GalNAc-specific lectin HAA binds terminal GalNAc on Gd-IgA1 but not sialylated or galactosylated GalNAc; (ii) neuraminidase removes sialic acid from sialylated GalNAc, which then becomes available for HAA binding, and (iii) galactose on GalNAc is not affected by neuraminidase (Fig. 4*a*). Using Golgi-enriched preparations from IgA1-secreting cells from IgAN patients and healthy controls as the respective sources of ST6GalNAc-II and C1GalT1 enzymes (47, 49), we validated the approach (Fig. 4*b*, *columns 1–5*). Next, we used Golgi-enriched preparations from IgA1-secreting cells from IgAN patients that have high activity of ST6GalNAc-II and performed *in vitro* enzyme sialylation of Gd-IgA1. Then we subjected these pre-sialylated IgA1 proteins to galactosylation reactions with C1GalT1 in the Golgi-enriched preparations from IgA1-secreting cells from IgAN patients (Fig. 4*b*, *columns 6* and *7*; *p* < 0.001) or healthy controls (Fig. 4*b*, *columns 8* and *9*; *p* < 0.001). The analyses of the reaction products revealed that sialylation of Gd-IgA1 protein blocked the subsequent galactosylation (Fig. 4*b*, *columns 6–9*). However, the blockade by sialylation was not complete, as there was a decrease in HAA reactivity after C1GalT1 reaction compared with that before the C1GalT1 reaction (*p* < 0.01 for *columns 3 versus columns 7* and *9*). Thus, effective galactosylation of IgA1 *O*-glycans can be substantially reduced by premature sialylation, and “oversialylation” of GalNAc may be a mechanism that contributes to galactose deficiency of IgA1 *O*-glycans in patients with IgAN.

## DISCUSSION

Lectin binding assays have been extensively used to examine the glycosylation patterns of circulating glycoproteins with *O*-glycans. In IgAN patients, IgA1 but not C1 inhibitor or IgD displays increased reactivity with several lectins specific for terminal GalNAc, indicating a decreased presence of galactose (24–26, 28, 29, 67, 68). These and other studies suggest that patients with IgAN exhibit galactose-deficient *O*-glycans uniquely on IgA1. Moreover, only a small subpopulation of IgA1 molecules is affected, and only some *O*-glycans are galactose-deficient (33, 37). As the hinge-region *O*-glycans of the IgA1 in the glomerular deposits of IgAN patients is galactose-deficient (34, 35), this altered glycosylation pattern plays a central role in the pathogenesis of IgAN (25).

We have shown that Gd-IgA1 is produced by IgA1-secreting cells due to dysregulation of several enzymes in the *O*-glycan biosynthetic pathway (47). An elevated serum level of Gd-IgA1 is a heritable trait, as many first-degree relatives of IgAN patients are frequently affected (30, 69, 70). However, these relatives usually manifest no clinical evidence of renal disease, suggesting that another factor(s) is needed to trigger development of IgAN (71).

Such a factor may be anti-glycan antibodies that bind Gd-IgA1 to form immune complexes (26, 36, 38). These immune complexes in the circulation have a large molecular mass and activate cultured human mesangial cells to proliferate and overproduce cytokines, chemokines, and components of extracellular matrix (41–44, 72–77). Some of these cytokines may injure podocytes to induce proteinuria (45, 46, 78). These observations led to a multi-hit autoimmune hypothesis for the pathogenesis of IgAN (33, 37, 38). Some or all of these steps are regulated genetically (79–84). We have shown that serum levels of Gd-IgA1 and anti-Gd-IgA1 antibodies (IgG and IgA1) predict clinical progression in patients with IgAN, thus confirming the role of autoantigen (Gd-IgA1) and autoantibodies in IgAN (85, 86).

The features of IgAN initially manifest most frequently in adolescents and young adults. Asymptomatic proteinuria and hematuria are characteristic clinical presentations (2, 7, 8). Notably, macroscopic hematuria often coincides with a mucosal infection, most commonly in the upper respiratory tract or digestive system (5, 87, 88). There is increasing evidence that mucosal innate immune defenses in IgAN patients exhibit genetic abnormalities (83, 89). These mucosal infections may alter the cytokine milieu for B cells and immunoglobulin-producing cells, including those secreting IgA1, and IgAN patients have elevated serum levels of TNFα and IL-6 (52). To confirm these previous observations, we measured circulating levels of IL-6 and IL-4 in a small cohort of IgAN patients and controls (Table 1), all without clinical signs of infection for at least 4 weeks before sampling. The IgAN patients had elevated levels of IL-6 (*p* < 0.01), but not of IL-4, compared with controls (data not shown). Thus, we tested whether IL-6 and/or some other cytokines may enhance production of Gd-IgA1 by using IgA1-secreting cells derived from the circulation of patients with IgAN and healthy controls.

**TABLE 1:** **Clinical characteristics of the study population** Data are expressed as the mean ± S.D. ND, not detected; RBC/HPF, red blood cells per high power field; UP/Cr, urinary protein/creatinine ratio.

	Age	Gender	Serum creatinine	UP/Cr	Hematuria
	*Year*	*M:F*	*mg/dl*	*g/g*	*RBC/HPF*
IgAN patients (*n* = 23)	32.5 ± 9.2	11:12	0.9 ± 0.3	0.6 ± 0.6	39.5 ± 27.2
Healthy controls (*n* = 11)	32.9 ± 4.4	4:7	0.6 ± 0.1	ND*^a^*	ND

*^a^* Protein concentration was below the limit of detection.

Our data revealed that IL-6 was the most active of the tested cytokines to affect glycosylation of IgA1. Treatment of cultured IgA1-secreting cells increased production of Gd-IgA1 and the degree of its galactose deficiency for IgAN patients and healthy controls. However, the greater degree of galactose deficiency of IgA1 secreted by the cells from healthy controls after IL-6 stimulation was less than that of IgA1 secreted by the cells from IgAN patients without IL-6 stimulation. This observation is consistent with an earlier publication that showed that IgA1 *O*-glycosylation normally varies in different immune responses and that patients with IgAN produce the full spectrum of IgA1 *O*-glycoforms (90). In the present study, IL-6 regulated IgA1 *O*-glycosylation. Notably, IL-6 is produced primarily at sites of acute and chronic inflammation and exerts an important regulatory effect on local immunoglobulin secretion. For example, epithelial cells increase IL-6 concentration to a much higher degree locally than what is observed in the circulation (64, 91). The amounts of IL-6 used in our assays were comparable to those produced by intestinal epithelial cells stimulated *in vitro* with LPS (64). As IL-6 is the major cytokine affecting immunoglobulin-producing cells and the terminal differentiation of plasma cells (92–94), it may regulate mucosal immune responses at multiple levels, including post-translational modifications of the secreted IgA1.

In our study, IL-4 was the only other tested cytokine that increased galactose deficiency of IgA1 *O*-glycans, but it did so for the cells from only IgAN patients. Previously, IL-4 was shown to increase galactose deficiency of *O*-glycans of IgA1 secreted by the Dakiki cell line through down-regulation of *Cosmc* and *C1GalT1* expression (54). We found that the cells from IgAN patients exhibited similar responses to IL-4. Notably, none of the cytokines tested in our experiments significantly increased the galactose content of IgA1 *O*-glycans. Thus, cytokines may regulate glycosylation of IgA1 but apparently only unidirectionally.

We have previously determined that the cells from IgAN patients secrete mostly polymeric IgA1, whereas cells from healthy controls secrete mostly monomeric IgA1 despite similar expression levels of J chain. Galactose deficiency affects preferentially polymeric rather than monomeric IgA1 (47). Therefore, we tested whether the increase in Gd-IgA1 is associated with greater representation of polymeric forms of IgA1 after treatment of the cells with a cytokine. No such change was observed.

Next, we determined whether the cytokines affected expression of genes encoding the key enzymes for the glycosylation of IgA1. IL-4 and IL-6 decreased expression of *C1GALT1* and *COSM*, and IL-6, but not IL-4, increased expression of *ST6GALNAC2*. The changes in enzymatic activities were proportional to the increases in galactose deficiency of IgA1.

**FIGURE 5.**
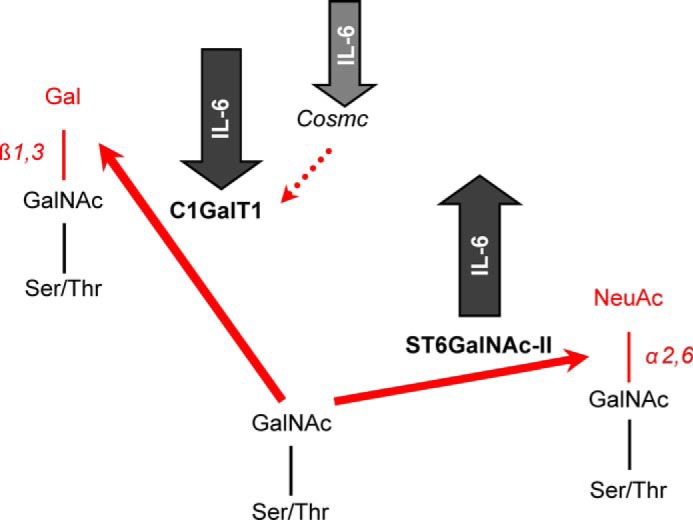
**IL-6 affects activity of key enzymes involved in *O*-glycosylation of IgA1 in IgAN.** IL-6 increases expression and activity of *ST6GALNAC2-*encoded ST6GalNAc-II and decreases expression and activity of *C1GALT1*-encoded C1GalT1 and expression of *COSMC*.

To directly verify the proposed roles of C1GalT1 and ST6GalNAc-II in production of Gd-IgA1, we used siRNA knockdown technology. Knockdown of either gene (*C1GALT1* or *ST6GALNAC2*) affected IgA1 *O*-glycosylation; *C1GALT1* knockdown increased the degree of galactose deficiency in both cell types, whereas *ST6GALNAC2* knockdown decreased galactose deficiency in the cells from only IgAN patients. This finding is consistent with the proposed role of overexpression of *ST6GALNAC2* leading to premature sialylation of GalNAc, thus preventing galactosylation (47, 95). To test this hypothesis, we assessed whether sialylation of Gd-IgA1 can block subsequent galactosylation. Our experiments confirmed that Gd-IgA1 sialylated at GalNAc is not effectively galactosylated. These experiments thus directly confirmed involvement of both glycosyltransferases in production of Gd-IgA1 and highlighted two possible pathways leading to galactose deficiency of IgA1 *O*-glycans, as outlined in Fig. 5. Notably, the results of siRNA knockdown experiments suggested that at normal levels of *ST6GALNAC2* expression the encoded enzyme does not contribute to premature sialylation of IgA1 *O*-glycans. It remains to be determined whether elevated expression of *ST6GALNAC2* may lead to abnormal localization of the enzyme in Golgi apparatus, as described for *ST6GALNAC1* overexpressed in cancer cells producing glycoproteins with sialylated GalNAc (96).

In summary, we found that a common cytokine, IL-6, not only can increase the IgA1 synthesis but also accentuate the degree of galactose deficiency on IgA1. Stimulation of IgA1-secreting cells from IgAN patients with IL-6 enhanced an already elevated activity of the GalNAc-specific sialyltransferase encoded by *ST6GALNAC2* gene, leading to increases in sialylation of terminal GalNAc. These changes, combined with IL-6-driven decreases in expression of *C1GALT1* and *COSMC* genes contributed to greater synthesis of Gd-IgA1 with sialylated or terminal GalNAc. Together, these results revealed an inherently abnormal regulation of intracellular enzymatic pathways of IgA1-secreting cells from IgAN patients and identified potential targets for future disease-specific therapy of IgAN. Future studies are needed to elucidate details of IL-6 signaling in IgA1-producing cells from IgAN patients.

## Footnotes

3 The abbreviations used are:

IgAN: IgA nephropathy
C1GalT1: core 1 β1,3-galactosyltransferase
Gd-IgA1: galactose-deficient IgA1
HAA: lectin from *H. aspersa*
ST6GalNAc-II: α-*N*-acetylgalactosaminide α-2,6-sialyltransferase 2.
